# Resilience, job satisfaction, intentions to leave nursing and quality of care among nurses during the COVID-19 pandemic – a questionnaire study

**DOI:** 10.1186/s12913-023-09648-5

**Published:** 2023-06-14

**Authors:** Saija Sihvola, Anu Nurmeksela, Santtu Mikkonen, Jaana Peltokoski, Tarja Kvist

**Affiliations:** 1 Faculty of Health Sciences, Department of Nursing Science, University of Eastern Finland, Kuopio Campus, Yliopistonranta 1 C, Canthia, P.O. Box 1627, Kuopio, FI-70211 Finland; 2 Faculty of Science and Forestry, Department of Applied Physics, University of Eastern Finland, Kuopio Campus, Yliopistonranta 1 C, Canthia, P.O. Box 1627, Kuopio, FI-70211 Finland; 3 Information Management, Wellbeing Services County of Central Finland, Viitaniementie 1, Jyväskylä, 40720 Finland

**Keywords:** COVID-19 pandemic, Intention to leave, Job satisfaction, Nurses, Resilience, Quality of care

## Abstract

**Background:**

The COVID-19 pandemic has challenged nurses and healthcare systems globally and raised major concerns for nurses’ wellbeing and working conditions. This cross-sectional and correlational study design aims to describe nurses’ resilience, job satisfaction, intentions to leave and quality of care, and explain their relationships during the COVID-19 pandemic.

**Methods:**

Data were collected from Registered Nurses (N = 437) in Finland through an electronic survey between February 2021 and June 2021. The questionnaire covered background characteristics (seven questions), resilience (four questions), job satisfaction (one question), intention to leave nursing (two questions), quality of care (one question), and requiring factors of the work (eight questions). The background variables and dependent variables were analyzed and presented using descriptive statistics. Structural equation modeling was used to explain dependent variables relationships. The study followed procedures recommended in the STROBE Statement for cross-sectional studies in efforts to maximize the quality of reporting results.

**Results:**

The surveyed nurses evaluated their resilience by mean score 3.92, more nurses had considered leaving nursing during the pandemic (16%) than before (2%). Nurses´ mean score of requiring factors of the work was 2.56 and overall job satisfaction 5.8. Structural equation modeling revealed that resilience affected their job satisfaction, which also influenced the quality of care, that was rated moderate (7.46 out of 10). Structural equation modeling indices of goodness of fit were NFI = 0.988, RFI = 0.954, IFI = 0.992, TLI = 0.97, CFI = 0.992, and RMSEA = 0.064. No direct relationship was found between resilience and intention to leave nursing.

**Conclusions:**

Good resilience promoted delivery of high-quality care by nurses during the pandemic and enhanced their job satisfaction, which reduced their intention to leave nursing. The results indicate that it is important to develop interventions that support nurses’ resilience.

**Impact:**

The study highlights the importance of nurses’ resilience during the pandemic, while job satisfaction may decrease and requiring factors of the work increase. Given the number of nurses who consider leaving nursing, there are clear needs to develop effective strategies to maintain quality healthcare with resilient, committed nursing staff.

## Introduction

The COVID-19 pandemic has been stressful for healthcare systems, especially for the health care professionals [1–5]. The pandemic demands crisis management and good nursing leadership [3]. In addition, to meet associated pressures both nurses and their healthcare organizations require strength and strategies that improve resilience, i.e., healthy coping skills and confidence for the future [6].

Nurses’ resilience is defined as a process [7, 8], involving both individual and external resources, that provides nurses with the ability to confront stressors and use the available resources in a way that helps them manage to cope with difficult situations [2, 7, 9].

It has been shown that lower resilience [10] and extreme workload [11] during the COVID-19 pandemic have been associated with higher intentions to leave nursing [10, 11], and that organizational support plays an important role in resilience [10] and job satisfaction [12]. Furthermore, previous studies have shown that resilience is negatively associated with anxiety [2], secondary traumatic stress, and burnout [9]. Thus, major concerns during the pandemic have included nurses’ resilience and wellbeing [13], and establishment of effective strategies to retain nurses in their positions and sustain future healthcare [10, 11].

### Background

Resilience has attracted researchers’ interest in various contexts for several decades, but nurses’ resilience is still a relatively new research area [7]. However, nurses´ intention to leave their profession is not a new phenomenon [14], and their job satisfaction has been a common topic for more than a decade [15–18]. Excessive work demands [11], an unfavorable work environment [19], low resilience [10], mental burden [11] and job dissatisfaction [16] are all associated with intentions to leave the profession [10, 11, 16, 19], and the pandemic has exacerbated the problems [10, 11].

During the pandemic the quality of care, especially in terms of missed care, has declined according to nurses’ assessments [20]. Insufficient supervision, support, and time, together with non-nursing duties, reportedly influence quality of care [21]. Nurses have been under continuous pressure, overworked, and skipping their breaks. Studies have shown that during the pandemic waves several nurses have had symptoms of extreme exhaustion, psychological distress, and job stressors. Exhaustion has not been limited to nurses’ work time, they have also had serious symptoms in their spare time [22], such as difficulties in sleeping [1, 22]. Nurses’ work is typically shiftwork, which itself can cause exhaustion, and it is important to recover between the work shifts [22]. Successful combination of work and personal life is also an important element of nurses’ job satisfaction [15]. However, a survey during early stages of the COVID-19 pandemic, in June 2020, showed that several nurses had symptoms of anxiety and depression [1].

Resilience provides protection for nurses from anxiety [2], secondary traumatic stress, and burnout [9]. Previous research has shown that it is associated with job satisfaction [23, 24], nurses’ work engagement [23], and quality of life [25]. It also increases nurses’ intention to remain in nursing [10, 26], and is an important factor for safe and quality care [27].

Crisis management and good nursing leadership are required during the pandemic [3]. Moreover, health services and public programmes should build resilience, not just react to health threats [28]. Thus, this study aims to describe nurses’ resilience, job satisfaction, intentions to leave and quality of care, and explain their relationships during the COVID-19 pandemic. Based on previous research, we hypothesized that job satisfaction, intention to leave nursing, and quality of care are all associated with resilience.

## The study

### Aim

The aim of this study was to describe nurses’ resilience, job satisfaction, intentions to leave, and quality of care and explain their relationships during the COVID-19 pandemic.

### Design

We employed a cross-sectional and correlational study design, following recommended procedures in the STROBE Statement for cross-sectional studies in efforts to maximize the quality of reporting results.

### Sample/Participants

Finland has 74 672 registered nurses in total [29]. Thus, according to a statistical power calculation, the purpose was to have a total of 383 answers (confidence interval 95%, p = .05), which was exceeded. The sample consisted of voluntarily registered nurses (N = 437). They were recruited via two Finnish hospitals, a university hospital and a central hospital (195 and 131 respondents, respectively), and the Finnish Nurses Association (111 respondents).

### Data collection

Data was collected in Finland through an electronic survey between February 2021 and June 2021. The participants were recruited via the study organizations that sent an email letter to registered nurses in which the research was presented and the link to the survey provided.

### Measures and their validity and reliability

The questionnaire included 23 questions covering: background characteristics (seven questions), resilience (four questions), job satisfaction (one question), intention to leave nursing (two questions), quality of care (one question), and requiring factors of the work (eight questions). The hypothesis underlying the study (job satisfaction, intention to leave nursing, and quality of care are all associated with resilience) was based on earlier literature when lower resilience [10] and extreme workload [11] have been shown to be related with greater intention to leave nursing [10, 11], association between resilience and job satisfaction have been found [23, 24] and negative connection between resilience and anxiety [2], secondary traumatic stress, as well as resilience and burnout [9] have been shown. In previous studies, resilience had a positive effect on nurses’ work engagement [23] and quality of life [25], and it supported nurses’ intention to retain nursing [10, 26].

#### Background characteristics

The background characteristics (seven items) were age, gender, working unit, experience in years as a nurse, experience in years in the current unit, and whether nurses had been taking care of patients with COVID-19 disease (daily, weekly, sometimes, not at all), and their vaccination status against the COVID-19-causing virus (first vaccination, two vaccinations, not any yet, not going to take any).

#### Resilience measures

Resilience was assessed with four items, based on prior literature, intended to capture nurses` resilience levels in terms of their confidence for the future, ability to confront stressors, ability to survive in difficult situations with good problem-solving skills, and perseverance [6–9].

The items were: *I trust in a better future*, *I identify and can utilize my personal strengths and ways which help me to survive in difficult situations*, *I have good problem-solving ability*, and *I have high perseverance*. They invited responses on a 5-point Likert scale (1 = not true at all, 2 = seldom true, 3 = sometimes true, 4 = often true, 5 = true nearly all the time). Scores of 1.00–2.49, 2.50–3.49, and 3.50–5.00 were categorized as indicative of low, moderate and good levels of resilience, respectively. A Cronbach alpha’s coefficient of 0.77 was obtained for the resilience scale.

#### Requiring factors of the work

The requiring factors of the work were addressed by eight items of a subscale of the Kuopio University Hospital Job Satisfaction Scale (KUHJSS), based on prior attempts to capture evaluations of the work demands, such as workload, staffing levels, feelings of stress, and working hours [15]. The eight questions also invited 5-point Likert-type responses (1 = totally disagree, 2 = partially disagree, 3 = I cannot say, 4 = partially agree, 5 = totally agree), with lower scores reflecting higher demands of the work. The scale´s Cronbach´s alpha coefficient was 0.84 in this study, indicating high reliability and consistent with previously reported values, which have ranged from 0.64 to 0.95 [15, 18].

#### Overall job satisfaction, quality of care, and intention to leave nursing

Based on previous studies, overall job satisfaction [30] and quality of care [30, 31] were evaluated with single questions using a scale of 0–10 (0 = worst and 10 = best). The question concerning the overall job satisfaction was: *How do you evaluate your overall job satisfaction during the COVID-19 pandemic?* The question of Quality of care was: *How do you evaluate quality of the care in your working unit during the COVID-19 pandemic?* Based on earlier research [32] intention to leave nursing was evaluated with single items: *How often did you consider leaving nursing before the COVID-19 pandemic? How often have you considered leaving nursing during the COVID-19 pandemic?* [32]. The items invited responses on a 5-point Likert scale (1 = daily, 2 = weekly, 3 = monthly, 4 = seldom, 5 = never at all).

### Ethical considerations

The study was approved by the University of Eastern Finland Committee on Research Ethics (Statement 2/2021). Permission for the research was given by the Finnish Nurses Association and participating hospitals. All analyses were carried out, and methods were used in accordance with the relevant guidelines and regulations (declaration of Helsinki). All the participants had to give informed consent, before answering the questionnaire.

### Data analysis

Scores for the background variables (seven items) and dependent variables (*resilience*, *requiring factors of the work*, *job satisfaction*, *intention to leave nursing*, and *quality of care)* were analyzed and presented using descriptive statistics (frequencies, percentages, mean scores, and standard deviations). Mean scores for resilience (four items) and requiring factors of the work (8 items), were calculated at both item and scale level, with possible ranges for both of 1–5. Overall job satisfaction (one item, range 0–10) and quality of care (one item, range 0–10), and intention to leave before and during the COVID-19 pandemic (two items, range 1–5) were all calculated at item level.

The significance of relationships between background variables and resilience, job satisfaction (requiring factors of the work), intention to leave nursing, and quality of care was assessed by Kruskal-Wallis tests. Pearson´s correlation analysis was used to evaluate the relationships between resilience and requiring factors of the work, overall job satisfaction, intention to leave nursing and quality of care. The internal consistency of the resilience scale and Requiring factors of the work was assessed by calculating Cronbach’s alpha values.

Structural equation modeling was used to test the relationships among the variables. Various goodness-of-fit measures were calculated to confirm the model’s fit. In the structural equation modeling (SEM), Normed Fit Index (NFI > 0.90), Relative Fit Index (RFI > 0.90), Incremental Fit Index (IFI > 0.90), Tucker-Lewis Index (TLI > 0.90), and Comparative Fit Index (CFI > 0.95) were used to evaluate the adequacy of the model. Additionally, the Root Mean Square Error of Approximation (RMSEA < 0.10) was calculated to estimate the approximation error due to model simplification. Standardized regression weight (*β*) estimates and standardized total effect (S.T.E.) values were compared to investigate the significance of relationships between variables [33]. For these analyses we used IBM SPSS Statistics for Windows, Version 27 (IBM Corp., Armonk, New York) and AMOS (version 27.0; IBM Corporation, Armonk, NY). In all these analyses, p ≤ .05 was regarded as statistically significant.

## Results

### Respondent characteristics

In total, 437 registered nurses consented to participate and completed the questionnaire. The age of the participants ranged between 21 and 69 years (mean 42.2 ± 11.0 years). Most (87.2%) were female, and their mean working experience as a nurse and at the current unit were 14.2 ± 10.6 and 8.2 ± 8.3 years, respectively, with ranges of 0–41 and 0–36 years, respectively. Most of the nurses (70%) had received either one or two vaccinations against the coronavirus SARS-CoV-2.

### Nurses´ resilience, requiring factors of the work, overall job satisfaction, intentions to leave nursing, and quality of care

Mean scores for the resilience and the requiring factors of the work scales (range 1–5) of the participating nurses (N = 437) were 3.92 ± 0.60 and 2.56 ± 0.86, respectively, and their mean scores for overall job satisfaction (range 0–10) and quality of care (range 0–10) were 5.80 ± 2.23 and 7.46 ± 1.71, respectively.

Ranges of mean scores for the resilience and requiring factors of the work items were 3.79-4.00 and 1.51–3.49, respectively. The items that obtained the highest and lowest mean scores on the resilience scale concerned nurses´ evaluations of their perseverance (Item 4, Mean 4.0 ± 0.79), and trust in the future (Item 1, Mean 3.79 ± 0.84). The highest and lowest scoring items on the requiring factors of the work scale concerned nurses´ satisfaction with their working hours and appropriateness of their salary in relation to demands of their work (Item 4 and 7: Mean 3.49 ± 1.27 and 1.51 ± 0.99, respectively). According to their responses, 16% (n = 70) of the nurses had considered leaving nursing daily during the pandemic, and 2% (n = 9) before it (Table 1).

**Table 1:** Frequencies, percentiles, mean item scores and standard deviations of resilience, requiring factors of the work, and intention to leave nursing (n = 437)

Scales, items						
**Resilience (n = 437)**	Not true at all	Seldom true	Sometimes true	Often true	True nearly all of the time	
	n (%)	n (%)	n (%)	n (%)	n (%)	Mean (SD)
1. I trust in a better future.	6 (1)	20 (5)	111 (25)	221 (51)	79 (18)	3.79 ± 0.84
2. I identify and can utilize my personal strengths and ways which help me to survive in difficult situations.	1 (0)	10 (2)	100 (23)	234 (54)	90 (21)	3.91 ± 0.78
3. I have good problem-solving ability.	3 (1)	3 (1)	94 (22)	236 (54)	101 (23)	3.98 ± 0.73
4. I have high perseverance.	3 (1)	12 (3)	83 (19)	222 (51)	117 (27)	4.00 ± 0.79
**Requiring factors of the work (n = 436)**	Totally disagree	Partially disagree	I cannot say	Partially agree	Totally agree	
	n (%)	n (%)	n (%)	n (%)	n (%)	Mean (SD)
1. My workload is appropriate.	82 (19)	186 (43)	6 (1)	114 (26)	48 (11)	2.68 ± 1.33
2. There is usually enough staff in my unit.	124 (28)	176 (40)	4 (1)	99 (23)	33 (8)	2.40 ± 1.31
3. I do not find my work too stressful.	84 (19)	195 (45)	16 (4)	102 (23)	39 (9)	2.58 ± 1.28
4. I am satisfied with my working hours.	32 (7)	105 (24)	14 (3)	188 (43)	97 (22)	3.49 ± 1.27
5. Combining work and personal life is successful.	44 (10)	140 (32)	13 (3)	165 (38)	74 (17)	3.20 ± 1.32
6. The workload is distributed evenly in my unit.	76 (17)	194 (45)	15 (3)	123 (28)	28 (6)	2.62 ± 1.24
7. My salary is appropriate in relation to the demands of my work.	312 (72)	79 (18)	3 (1)	32 (7)	10 (2)	1.51 ± 0.99
8. The upper management of the hospital district appreciates my work.	184 (42)	143 (33)	40 (9)	56 (13)	13 (3)	2.02 ± 1.14
**Intention to leave nursing (n = 437)**	Daily	Weekly	Monthly	Seldom	Never at all	
	n (%)	n (%)	n (%)	n (%)	n (%)	Mean (SD)
1. How often did you consider leaving nursing before the COVID-19 pandemic?	9 (2)	40 (9)	88 (20)	193 (44)	106 (24)	3.78 ± 0.99
1. How often have you considered leaving nursing during the COVID-19 pandemic?	70 (16)	127 (29)	85 (20)	81 (19)	74 (17)	2.91 ± 1.34

### Relationships between background characteristics and outcomes

Age, working unit, experience as a nurse, vaccination status, and taking care of patients with COVID-19 disease were all found to significantly affect the nurses’ resilience, job satisfaction, intention to leave nursing, and/or quality of care. No significant differences between gender differences in any of these outcomes were detected. Frequencies and percentages of the background characteristics and means and standard deviations of the study variables are presented in Table 2.

**Table 2:** Background characteristics and outcomes: frequencies, percentages, means and standard deviations

Background characteristics	N = 437	Resilience	Requiring factors of the work	Overall job satisfaction	Intention to leave nursing before the pandemic	Intention to leave nursing during the pandemic	Quality of care
	n (%)	Mean, SD	Mean, SD	Mean, SD	Mean, SD	Mean, SD	Mean, SD
**Age (n = 436)**							
≤ 30 years	84 (19)	3.90 ± 0.69	2.45 ± 0.80	5.33 ± 2.32	3.61 ± 1.02	2.63 ± 1.29	7.30 ± 1.64
31–40 years	115 (26)	3.93 ± 0.58	2.49 ± 0.89	5.50 ± 2.10	3.75 ± 0.94	2.75 ± 1.29	7.32 ± 1.51
41–50 years	114 (26)	3.88 ± 0.64	2.54 ± 0.78	5.69 ± 2.19	3.68 ± 1.11	2.81 ± 1.35	7.41 ± 1.90
51–69 years	123 (28)	3.96 ± 0.55	2.66 ± 0.89	6.35 ± 2.12	4.00 ± 0.94	3.27 ± 1.32	7.64 ± 1.75
Missing n (%)	1 (0.2)						
**Gender (n = 431)**							
Female	376 (87)	3.92 ± 0.59	2.54 ± 0.85	5.79 ± 2.19	3.81 ± 0.98	2.94 ± 1.33	7.44 ± 1.68
Male	53 (12)	3.93 ± 0.76	2.58 ± 0.86	5.69 ± 2.28	3.60 ± 1.25	2.60 ± 1.39	7.41 ± 1.94
Do not want to tell.	6 (1)	3.50 ± 0.35	2.00 ± 0.35	3.00 ± 1.41	3.00 ± 1.41	2.00 ± 1.41	7.00 ± 0.00
Missing n (%)	6 (1)						
**Working unit (n = 437)**							
Urgent care	42 (10)	3.91 ± 0.62	2.38 ± 0.83	5.63 ± 2.24	3.98 ± 1.05	3.11 ± 1.56	6.76 ± 1.91
Acute ward unit	128 (29)	3.83 ± 0.61	2.24 ± 0.70	5.24 ± 2.22	3.71 ± 1.08	2.75 ± 1.33	7.28 ± 1.82
Intensive care	65 (15)	3.96 ± 0.51	2.44 ± 0.85	5.37 ± 2.40	3.81 ± 0.96	2.70 ± 1.25	7.90 ± 1.55
Anesthesia and operative	40 (9)	3.62 ± 0.63	2.50 ± 0.69	5.35 ± 2.23	3.55 ± 0.95	2.58 ± 1.20	7.26 ± 1.97
Outpatient clinic	92 (21)	4.03 ± 0.61	2.96 ± 0.90	6.29 ± 1.93	3.83 ± 0.98	3.07 ± 1.33	7.85 ± 1.33
Other units	70 (16)	4.10 ± 0.60	2.78 ± 0.87	6.21 ± 2.07	3.80 ± 0.97	3.17 ± 1.31	7.19 ± 1.62
Missing n (%)	0 (0)						
**Experience as a nurse (n = 432)**							
≤ 1 year	26 (6)	4.19 ± 0.81	2.63 ± 0.84	6.27 ± 2.24	4.15 ± 1.01	3.38 ± 1.33	7.46 ± 1.70
2–5 years	88 (20)	3.83 ± 0.62	2.44 ± 0.82	5.35 ± 2.16	3.84 ± 0.96	2.77 ± 1.22	7.21 ± 1.68
6–10	88 (20)	3.90 ± 0.61	2.40 ± 0.88	5.31 ± 1.96	3.63 ± 0.88	2.48 ± 1.16	7.41 ± 1.52
11–20	107 (25)	3.86 ± 0.56	2.56 ± 0.87	5.64 ± 2.38	3.60 ± 1.04	2.83 ± 1.42	7.24 ± 1.92
≤ 21	123 (29)	3.99 ± 0.58	2.70 ± 0.83	6.40 ± 2.07	3.92 ± 1.05	3.28 ± 1.34	7.77 ± 1.63
Missing n (%)	5 (1)						
**Experience as a nurse in the current unit (n = 428)**							
≤ 1 year	91 (21)	4.07 ± 0.67	2.72 ± 0.88	5.97 ± 2.14	3.74 ± 1.08	3.00 ± 1.30	7.56 ± 1.55
2–5 years	127 (30)	3.90 ± 0.60	2.45 ± 0.88	5.43 ± 2.14	3.79 ± 0.98	2.68 ± 1.27	7.15 ± 1.73
6–10	90 (21)	3.84 ± 0.64	2.49 ± 0.80	5.74 ± 2.29	3.72 ± 0.93	2.86 ± 1.34	7.27 ± 1.95
11–20	78 (18)	3.89 ± 0.51	2.50 ± 0.79	5.78 ± 2.25	3.57 ± 1.12	2.74 ± 1.40	7.62 ± 1.68
≤ 21	42 (10)	3.89 ± 0.56	2.66 ± 0.88	6.38 ± 2.14	4.33 ± 0.69	3.68 ± 1.21	8.03 ± 1.25
Missing n (%)	9 (2)						
**Have taken care of patients with COVID-19 disease (n = 437)**							
Daily	8 (2)	4.19 ± 0.75	2.41 ± 0.67	4.63 ± 3.16	4.00 ± 1.31	2.75 ± 1.75	7.25 ± 1.58
Weekly	89 (20)	3.87 ± 0.60	2.25 ± 0.79	5.12 ± 2.12	3.87 ± 0.91	2.63 ± 1.31	7.23 ± 1.72
Sometimes	145 (33)	3.86 ± 0.62	2.50 ± 0.86	5.61 ± 2.31	3.74 ± 0.96	2.86 ± 1.36	7.32 ± 2.03
I have not taken care of them.	195 (45)	3.97 ± 0.60	2.72 ± 0.84	6.21 ± 2.01	3.75 ± 1.08	3.04 ± 1.30	7.61 ± 1.41
Missing n (%)	0 (0)						
**Vaccination against the COVID-19 virus (n = 436)**							
One vaccination	107 (25)	3.97 ± 0.63	2.71 ± 0.92	6.22 ± 2.11	3.79 ± 1.07	3.09 ± 1.41	7.53 ± 1.88
Two vaccinations	199 (46)	3.90 ± 0.56	2.45 ± 0.76	5.75 ± 2.11	3.85 ± 0.98	2.90 ± 1.33	7.54 ± 1.59
No vaccination	105 (24)	3.92 ± 0.61	2.67 ± 0.91	5.67 ± 2.22	3.72 ± 0.93	2.79 ± 1.25	7.35 ± 1.55
Not going to take vaccination	25 (6)	3.86 ± 0.85	2.13 ± 0.73	4.40 ± 2.63	3.36 ± 1.22	2.48 ± 1.29	6.56 ± 2.20
Missing n (%)	1 (0.2)						

#### Nurses´ resilience during the pandemic

Nurses who had one year or less of working experience as a nurse rated their overall resilience more highly on average than nurses with more experience (p = .007). In addition, nurses who worked in intensive care, urgent care, acute ward, or anesthesia and operative units rated their resilience less highly than those worked in an outpatient clinic or other units (p = .003) (Table 2).

#### Requiring factors of the work

Nurses who worked in urgent care, acute ward, intensive care, or anesthesia and operative units scored requiring factors of the work more highly on average than those who worked in an outpatient clinic or other units (p = .001). Their ratings of the requiring factors of the work were also significantly lower if they had over 20 years’ experience as a nurse than if they had less experience (p = .043). Similarly, nurses evaluated requiring factors of the work lower if they had not taken care of patients with a COVID-19 disease (p = .001) and had received the first vaccination or had not received any vaccination yet (p = .006) (Table 2).

#### Overall job satisfaction

Nurses older than 50 years rated their overall job satisfaction more highly on average than their younger colleagues (p = .002). Overall job satisfaction was scored less highly by nurses who worked in urgent care, acute wards, intensive care, and anesthesia or operative care units than those who worked in an outpatient clinic or other units (p = .031). Nurses evaluated their overall job satisfaction better if they had worked one year or less or more than 20 years as a nurse (p = .001), and if they had worked in their current unit for either one year or less, or more than 20 years (p = .034). Nurses rated their job satisfaction less highly if they had taken care of patients with a COVID-19 disease (p = .001). In addition, nurses who were not going to take a COVID-19 vaccination rated their job satisfaction as lower than those who had received one, or two vaccinations or answered that they had not yet received any (p = .004) (Table 2).

#### Intention to leave nursing before the COVID-19 pandemic

Before the COVID-19 pandemic, the oldest nurses (51–69 years) had significantly higher intention to remain in nursing than the younger nurses (p = .031). Moreover, intention to leave nursing before the COVID-19 pandemic was lower among participants who had worked for a year or less as a nurse than among more experienced colleagues (p = .002) or over 20 years in their current unit (p = .005) (Table 2).

#### Intention to leave nursing during the COVID-19 pandemic

Older age (51–69 years) was statistically significant factor to remain in nursing during the pandemic while younger nurses had greater intention to leave nursing (p = .003). Moreover, intention to leave nursing was lower among participants who had worked for a year or less, or over 20 years as a nurse (p = .001) and over 20 years in the current unit (p = .001) (Table 2).

#### Quality of care

Quality of care was scored more highly by nurses who worked in an intensive care unit or outpatient clinic than by those who worked in an urgent care, acute ward, anesthesia and operative, or other units (p = .001). It was also rated more highly by nurses who had worked in their current unit for over 20 years than by colleagues who had spent less time in their current unit (p = .011) (Table 2).

### Correlations between resilience, requiring factors of the work, overall job satisfaction, intention to leave nursing, and quality of care

Highly significant correlations were found between resilience, requiring factors of the work, overall job satisfaction, intention to leave nursing, and quality of care (p ≤ .001) (Table 3).

**Table 3:** Pearson´s correlations between resilience, requiring factors of the work, overall job satisfaction, intention to leave nursing and quality of care

Variable	Resilience	Requiring factors of the work	Overall job satisfaction	Intention to leave nursing before the COVID-19 pandemic	Intention to leave nursing during the COVID-19 pandemic	Quality of care
Resilience	1					
Requiring factors of the work	0.291***	1				
Overall job satisfaction	0.319***	0.613***	1			
Intention to leave nursing before the COVID-19 pandemic	0.205***	0.288***	0.290***	1		
Intention to leave nursing during the COVID-19 pandemic	0.276***	0.528***	0.585***	0.650***	1	
Quality of care	0.271***	0.422***	0.548***	0.315***	0.221***	1

***p ≤ .001

### Structural equation modeling

**Fig. 1:**
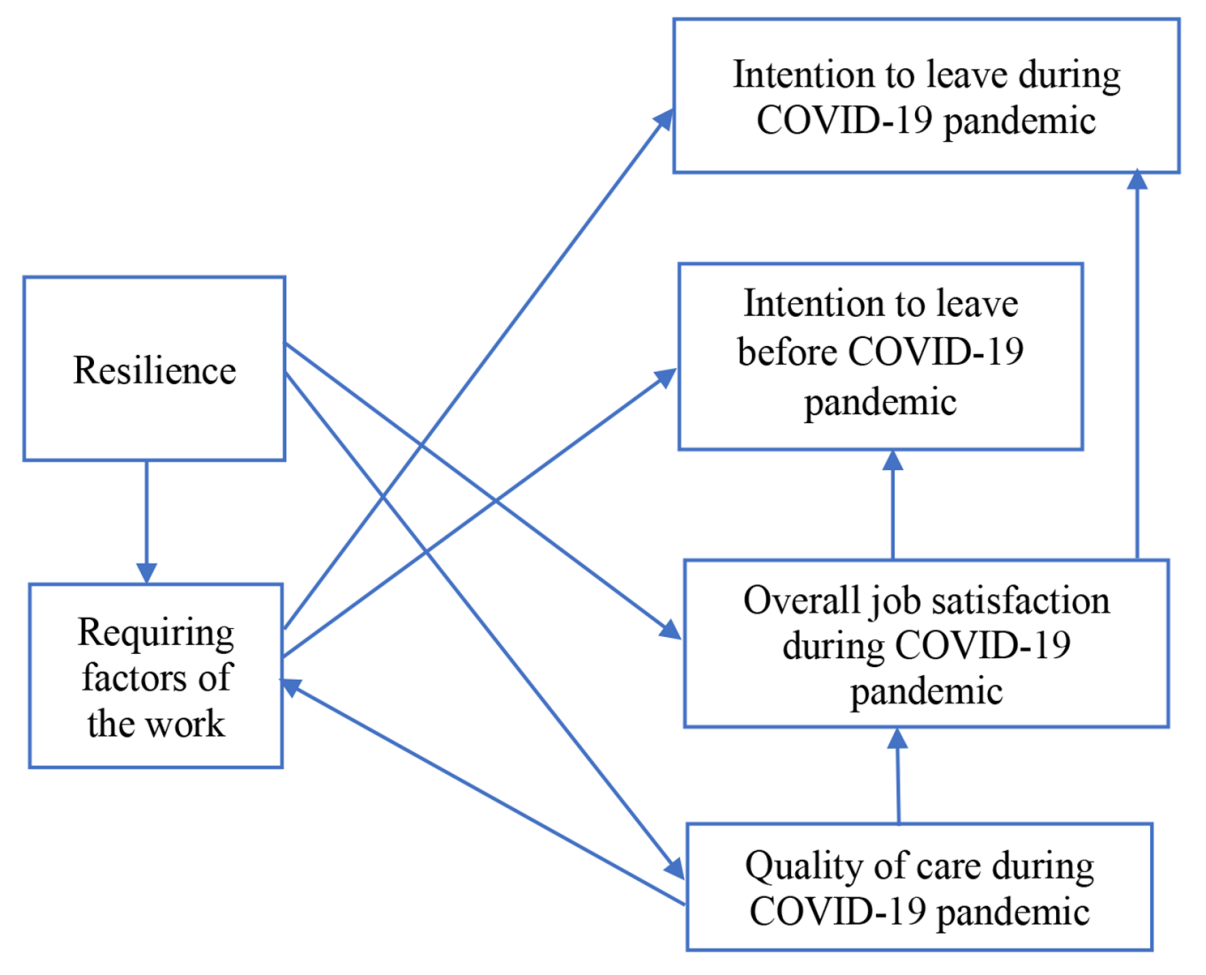
Structural equation modeling of variables of nurses’ Resilience and Requiring factors of the work, Intention to leave before and during the COVID-19 pandemic, and nurses’ estimated Overall job satisfaction and Quality of care during the COVID-19 pandemic

Results of the structural equation modeling (SEM) of relationships among the six studied variables are illustrated in Fig. 1. The overall model had satisfactory results for goodness of fit. Indices results were NFI = 0.988, RFI = 0.954, IFI = 0.992, TLI = 0.97, CFI = 0.992 and RMSEA = 0.064.

The acquired model revealed nine direct relationships between the variables. Magnitudes of the effects are shown in Fig. 1. Nurses’ resilience was related to their ratings of the quality of care during the COVID-19 pandemic (β = 0.270, p < .001), requiring factors of the work (β = 0.190, p < .001) and overall job satisfaction during the COVID-19 pandemic (β = 0.184, p < .001). In other words, when nurses rated their resilience higher, they also rated the quality of care more highly, requiring factors of the work less severely, and overall job satisfaction during the pandemic more highly (Table 4).

**Table 4:** Effects of the paths in the model - Standardized regression weight estimates (β), standardized total effects (S.T.E), regression weights (Est.), standard errors (S.E.), critical ratios (C.R.), probability values (p), and covariances between variables

			β	S.T.E	Est.	S.E.	C.R.	p
Quality of care	<---	Resilience	0.270	0.270	0.770	0.131	5.860	< 0.001
Requiring factors of the work	<---	Resilience	0.190	0.291	0.270	0.063	4.315	< 0.001
Overall job satisfaction	<---	Resilience	0.184	0.319	0.677	0.150	4.519	< 0.001
Overall job satisfaction	<---	Quality of care	0.500	0.500	0.648	0.053	12.317	< 0.001
Requiring factors of the work	<---	Quality of care	0.372	0.372	0.185	0.022	8.427	< 0.001
Intention to leave nursing before C-19	<---	Overall job satisfaction	0.183	0.183	0.082	0.026	3.189	0.001
Intention to leave nursing during C-19	<---	Overall job satisfaction	0.420	0.420	0.252	0.028	8.855	< 0.001
Intention to leave nursing before C-19	<---	Requiring factors of the work	0.175	0.175	0.204	0.067	3.058	0.002
Intention to leave nursing during C-19	<---	Requiring factors of the work	0.271	0.271	0.424	0.074	5.719	< 0.001
Covarying variables				**Covariances** **Est.**		**S.E.**	**C.R.**	**P**
Requiring factors of the work	<-->	Overall job satisfaction			0.660	0.073	9.030	< 0.001
Intention to leave job during C-19	<-->	Intention to leave job before C-19			0.597	0.055	10.844	< 0.001

Abbreviations: C-19 = COVID-19 pandemic

Nurses’ ratings of quality of care during the COVID-19 pandemic were positively related to their overall job satisfaction during the pandemic (β = 0.500, p < .001) and negatively related to their ratings of the severity of requiring factors of the work (β = 0.372, p < .001). Thus, those who rated the quality of care highly had less intention to leave nursing during the pandemic, and greater satisfaction with requiring factors of the work (Table 4).

Nurses’ evaluations of the overall job satisfaction during the COVID-19 pandemic were also negatively related to their intention to leave nursing during (β = 0.420, p < .001) and before the COVID-19 pandemic (β = 0.183, p = .001). (Table 4)

Moreover, nurses’ ratings of the requiring factors of the work were associated with intention to leave the job both during (β = 0.271, p < .001) and before (β = 0.175, p = .002) the pandemic; those who expressed strong dissatisfaction with the requiring factors of the work also expressed more frequent thoughts of leaving their job before and during the pandemic (Table 4).

Two indirect relationships confirmed the final model by two-way relations. Relationships between both nurses’ overall job satisfaction during the COVID-19 pandemic and the requiring factors of the work (p < .001), and intention to leave the job before and during the COVID-19 pandemic, were highly significant (p < .001) (Table 4).

## Discussion

The COVID-19 pandemic has challenged nurses and healthcare systems globally and raised major concerns for nurses’ wellbeing and working conditions [1–5, 10, 11, 25, 26]. We aimed to describe nurses’ resilience, job satisfaction, intentions to leave and quality of care during the COVID-19 pandemic and explain their relationships by using Structural equation modeling.

The findings of this study indicate that nurses´ resilience was at a good level. The results show that newly graduated nurses who had one year or less work experience had the highest resilience. However, substantial shares of the nurses rated requiring factors of the work as rather high, their overall job satisfaction as low, and the quality of care in their own working unit as moderate. Their intention to leave was higher during the pandemic than before it. During the COVID-19 pandemic, of the total 437 nurses, 45% reported daily or weekly intentions to leave nursing, compared to just 11% before the pandemic. Nashwan et al. (2021) reported similar findings. Nursesʼ intentions to leave increased significantly during the pandemic and stayed notably high during it. This is an important finding because nurses’ intentions to leave reflects something about their well-being, and the factors associated with their turnover intentions should be addressed constantly, particularly during crises [34]. Many of these reasons are connected to the nursing leadership, with Gensimore et al., (2020) suggesting that nurse leaderʼs visibility and actions are crucial in positive perception of the practice environment and influence nursesʼ intention to stay [31].

We found that nurses of the oldest age group had significantly higher overall job satisfaction and had less intention to leave nursing both before and during the pandemic than younger nurses. These results are consistent with findings by Koehler and Olds (2022). They found that Generation X and Baby Boomer nurses have greater engagement in work than Millennials. However, the most common reasons for nurses’ intention to leave their position are more likely preventable, such as payment, career advancement opportunities, and dissatisfaction with management, work schedules, and/or work environments [35].

The structural equation model confirmed our hypothesis that nurses’ job satisfaction, intentions to leave nursing, and quality of care are all associated with resilience. Resilience directly affected the ratings of job satisfaction and requiring factors of the work, in accordance with previous studies [23, 24]. Previous studies have also shown that resilience promotes nurses’ intention to remain in nursing [10, 26] and is an important factor for safe and quality care [27]. Similarly, we found that resilience had a positive effect on perceived quality of care, which had a positive effect on nurses’ overall job satisfaction. However, we detected no direct effect between resilience and intention to leave nursing. Participants’ low ratings of requiring factors of the work and high job satisfaction ratings were linked to low intentions to leave their job, before and during the pandemic. Previous studies have also shown that job satisfaction is an important factor for nurses’ engagement [24]. In addition, we found that nurses evaluated requiring factors of the work less severely when they had not taken care of COVID-19 patients.

Our results show that the COVID-19 pandemic has affected nurses’ wellbeing. Substantial shares of participants scored the requiring factors of their work as high, overall job satisfaction as low and had considered leaving their profession. For example, 28% responded that there were not usually enough staff in their working unit. They also evaluated the quality of the care in their current units as moderate. Previous study by Sasso et al. (2019) has shown that understaffing is related to nursesʼ intentions to leave their job. Moreover, in this study, most of the participants (n = 312, 72%) stated that their salary was not appropriate in relation to the demands of their work [16]. This is consistent with previous findings. For example, Jiang et al. (2019) reported that both salary and non-financial incentives, together with self-rated health and perceived respect from patients, are significantly positively associated with nurses’ job satisfaction [17].

However, some results of our study are encouraging, notably that the nurses had good levels of self-reported resilience, that emphasized their perseverance, problem-solving skills and abilities to utilize personal strength. Previous studies have shown that resilience protects nurses from mental illness [2, 9] and is associated with better quality of life [25]. An important finding of our study is that the oldest nurses were more satisfied with their job than their younger colleagues, and more frequently expressed a desire to remain in their profession. This may highlight strong commitment to their work, but it may also reflect something about their economic situation and limited possibilities to retire or obtain other employment. However, in a large study Wargo-Sugleris et al. (2018) identified job satisfaction as the main reason for aging nurses to stay at work and avoid early retirement [36]. A notable finding of our study is that younger nurses more frequently consider leaving nursing, which is a major concern for the future of healthcare.

### Strengths and limitations

This cross-sectional study provides an overview of the situation among nurses during the second wave of the COVID-19 pandemic in Finland 2021. However, it had several limitations. First, a small sample size (n = 437) limited to Finnish nurses restricts the international generalization of the results, although the sample included nurses working in various fields of nursing in several hospitals. Notably, a sample size of 200 should be sufficient for constructing small- or medium-sized SEM models, but the threshold size depends on the model’s complexity [33]. The full SEM we generated had goodness of fit with NFI, RFI, IFI, TLI, CFI and RMSEA values of 0.988, 0.954, 0.992, 0.97, 0.992 and 0.064, respectively. Second, the items measuring nurses’ resilience were created for this study and require more validation. However, the KUHJSS is a highly validated instrument, and Cronbach´s alpha values for the resilience and KUHSS items were acceptable (0.77 and 0.84, respectively) in this study. Finally, the data obtained using these instruments were self-reported, which can demonstrate a certain degree of bias in evaluations. However, to the best of our knowledge, this is the first study in Europe that addressed nurses’ resilience, job satisfaction, intention to leave nursing, and quality of care during the COVID-19 pandemic with an aim to describe their relationships together in the same study. The strength of this study is also that questions were well answered, as shown in the tables, only 0–2% of the responses were missing. Most of them were in background characteristics (missing n = 0–9), while all participants had answered questions concerning resilience, intention to leave nursing and overall job satisfaction and only one response was missing from requiring factors of the work and quality of care.

## Conclusions

Higher resilience levels (self-reported) among participating nurses promoted delivery of high-quality care by nurses during the pandemic and enhanced their job satisfaction, which reduced their intention to leave nursing. The results highlight the importance of developing interventions that support nurses´ resilience.

## Data Availability

The data are not publicly available due to them containing information that could compromise research participant privacy. Consent to share data publicly was not asked from the participants of this study but are available from the corresponding author on reasonable request.
